# Quinine Pills

**Published:** 1874-10

**Authors:** 


Pills of Sulphate of Quinia—Mr. A. Schreiber, of Tell City, Ind., informs us that a smaller quinia pill can be made by using a mixture of equal parts of glycerine and muriatic acid, than with the glycerine alone; Mr. S. has used such a mixture for this purpose for several years

